# Self-Reported Oral Hygiene Performance of Patients in Albania: A Questionnaire-Based Survey

**DOI:** 10.3390/dj13010001

**Published:** 2024-12-24

**Authors:** Blerina Zeza, Blerina Osmani, Erdita Cenameri, Busra Emir

**Affiliations:** 1 Department of Dentistry, Albanian University, 1001 Tiranë, Albania; 2 Independent Researcher, 1001 Tiranë, Albania; 3 Independent Researcher, 80337 Munich, Germany; 4 Department of Biostatistics, Faculty of Medicine, Izmir Katip Celebi University, 35620 Izmir, Turkey; busra.emir@ikcu.edu.tr

**Keywords:** interdental bleeding, interdental instruments, mouthrinse, oral hygiene, questionnaire, toothbrushing

## Abstract

**Background/Objectives:** Oral hygiene is a key factor for dental and periodontal diseases and the prognosis of any treatment to restore their consequences. The present survey aimed to evaluate how well informed patients in Albania are on oral hygiene, given the scarce evidence on this topic. **Methods:** This survey was performed using a Google Forms questionnaire on oral hygiene habits, type and technique of instruments used, and frequency of dental recall visits. **Results:** A total of 1006 questionnaires were filled in a one-month time period (January 2020) from a population of 75.2% females and 24.8% males, 32.7 ± 11.3 years of age, from whom 94.6% reported a lack of systemic diseases and 80% were non-smokers. All of the population brushed their teeth at least once daily. The most used type of toothbrush was a manual one (91.1%) with medium-bristle hardness in 71% of cases. The most prevalent way of using the toothbrush was through circular movements (36.7%) with a duration of 2–3 min in 72% of the population. Only 77.1% of the population used interdental instruments, mainly interdental floss. **Conclusions:** Within the limits of this study, it could be concluded that the present population seemed to be mostly appropriately informed, but the information needs further revision and promotion by dentists.

## 1. Introduction

Dental caries and periodontal diseases are among the most prevalent oral diseases, with a high global economic impact that increased the total burden by 37% for untreated caries and by 67% for severe periodontitis due to global population growth and increased tooth retention [1]. Dental plaque biofilm is a major biological determinant common to the development of both diseases and even biological complications after tooth extraction due to suture retention [2,3,4]. Dental caries is defined as a localized chemical dissolution of the tooth surface caused by acid production by the dental biofilm (dental plaque) exposed frequently to sugars [5], whereas periodontal diseases (gingivitis and periodontitis) are inflammatory diseases, with the most important risk factor the accumulation of a dental plaque biofilm at and below the gingival margin, which is then associated with an inappropriate and destructive inflammatory immune response [6]. Thus, the most important behavioral factor, affecting both dental caries and periodontal diseases, is routinely performed oral hygiene with toothpaste either by the individuals themselves or by caregivers [7].

A periodontal treatment plan includes instructions and motivation on oral hygiene, not only for the primary prevention of the establishment of disease but also for the secondary prevention after the diseases have been actively treated [8]. Dental plaque biofilm is related to the establishment of periodontal inflammation, as demonstrated by the group of Löe et al., 1965, with the complete interruption of any oral hygiene measures for 21 days being associated with the establishment of gingivitis in all participants; but, once the dental plaque is removed, it leads to complete resolution of gingivitis [9]. However, in case the patient refrained from any oral hygiene measures and instructions, 90% of them could develop periodontitis, the irreversible form of periodontal inflammation [10]. In these cases, the reinstitution of oral hygiene cannot replace the active treatment, which is based on the gravity and severity of the disease and could include periodontal surgeries or tooth extraction. However, performing periodontal surgeries in conditions where the inclusion criteria related to inflammation and oral hygiene level are not fulfilled may compromise the success of the treatment [11]. Furthermore, performing periodontal surgeries in patients who do not follow a supportive periodontal therapy (SPT) to maintain an acceptable level of oral hygiene could lead to further bone loss around treated teeth, and this bone loss has been estimated to be 3–5 times more pronounced that the natural progression of bone loss in periodontitis [12].

The dental plaque biofilm does present a key element in oral diseases and in general health conditions as well. The link between poor oral health and systemic diseases (cardiovascular diseases, poor glycemic control in diabetics, low birth weight, preterm babies, and a variety of other conditions) has been increasingly recognized, showing an association, although it is not always strong [13,14,15,16].

In the 2022 WHO Oral Health Albania Profile, it was reported that there was a prevalence of 39.8% for untreated caries of permanent teeth in individuals over 5 years old and a prevalence of 14.9% for severe periodontal disease in individuals over 15 years old [17]. The estimated global average prevalence of caries of permanent teeth and severe periodontitis are 29% and 19%, respectively [18]. There is little evidence of periodontal disease and oral hygiene in Albania. In 2010, Hysi et al. reported that only 67% of 12-year-old children in Albania brushed their teeth at least once a day, and 87% consumed sweeties every day, emphasizing the need for dental counseling and education to improve oral health [19]. A survey of 104 children 11–15 years old showed that gingivitis was present in 96% of them and was associated with the presence of dental plaque, despite them reporting toothbrushing daily [20]. Among 1889 adolescents (16–19 years old) in Albania, only 43.9% of them had a plaque index of 0, which highlighted again the need for preventive care programs to improve oral health conditions and reduce oral pathology risk factors in Albania [21]. However, well-performed oral hygiene requires appropriate informing of the patient and appropriate execution of instructions by the patient. Oral hygiene information can be obtained from dental professionals through direct communication or on the internet. In the present country context where the dental hygienist professional is not well integrated into everyday practice, oral hygiene instructions and motivation are mostly provided by dentists, which evidence reported could influence the oral hygiene information transmission to the patient [22]. On the other hand, the information provided by the internet mostly, in YouTube videos, had been valued as a need to improve the quality of the content [23]. The aforementioned results show that there is a need for improvement in performing oral hygiene. However, well-performed oral hygiene requires appropriate informing of the patient and appropriate execution of instructions by the patient.

In view of the current evidence and the importance that appropriate information on oral hygiene has on performing oral hygiene daily, the aim of the present survey was to evaluate how well informed patients in Albania are on oral hygiene.

## 2. Materials and Methods

This is an observational study performed through a questionnaire distributed by dentists and pharmacists to their patients to be filled out manually or electronically. The distribution was extended for one month in January 2021. The electronically distributed questionnaire was generated from Google Forms. The authors declare that the investigation was carried out following the rules of the Declaration of Helsinki of 1975, revised in 2013, and was approved by the Institutional Review Board (Ethic Committee) of Albanian University (protocol code 1025 and date of approval 24 December 2020).

The questions were formulated based on the literature [24] and authors’ clinical experience and adapted in a way that each one of them would be easily understood, despite the participants’ level of information, and it underwent a prior internal validation within a closed small group of people with whom it was possible to exchange impressions and suggestions on the clarity and relevance of each question, followed by modifications made to improve the questionnaire.

The validated questionnaire included an introductory paragraph that served as informed consent as well. It gave a brief description of the aim of this study and the privacy respect and usage of the data gathered at the end of the survey, and it emphasized the free will decision of the participants to continue the completion of the questionnaire without any consequences. Participants were explained that demographic data would be used only in relation to the purpose of this study and were encouraged to spread it among relatives and acquaintances.

The introductory paragraph was followed by questions related to the topic of this study. The questions were mainly closed ones, giving patients the possibility to identify more correctly the answer to the question, as long as it was assumed that participants not necessarily would have proper dental and medical knowledge. The first questions aim at collecting demographic information about age, gender, the city where they live, the presence of systemic disease (if the subject was aware of it), smoking, and if they have ever been informed by their dentist of periodontal diseases in relation to oral hygiene. The following questions, aiming at collecting information on oral hygiene, were grouped into questions regarding toothbrushing (type of toothbrush, hardness of toothbrushing bristles, technique, frequency and duration of toothbrushing, toothbrushing after certain fruits, and frequency of toothbrush change), interdental hygiene (type of interdental instrument, frequency of use, bleeding after use), and chemical plaque control (toothpaste and mouth rinse).

### Statistical Analysis

Albania has a population of 2.8 million individuals, with almost one-third living in the capital [25]. The sample size was determined using a priori power analysis with G*Power (version 3.1.9.7). Using correlation analysis as the statistical method, it was determined that a minimum of 779 participants should be included in this study to achieve an effect size of 0.10 (small), a type 1 error of 0.05, and a minimum power of 0.80.

The data were extracted from Google Forms in an Excel file and adjusted for statistical analysis. All statistical analyses were performed using the IBM SPSS Statistics 26.0 package 91 program (IBM Corp., Armonk, New York, NY, USA). Descriptive statistics were given as number of units (*n*), percentage (%), mean ± standard deviation, median, and interquartile range values. The normal distribution of the data of quantitative variables was evaluated with the Shapiro–Wilk normality test and Q-Q graphs. Comparisons between groups for continuous variables were evaluated with the Mann–Whitney U test if the data were not normally distributed. Comparisons of independent groups with more than two subcategories were evaluated with a Kruskal–Wallis analysis according to the distribution of data normality test results. If there was a difference as a result of the Kruskal–Wallis analysis, Dunn’s Bonferroni test was used as a multiple comparison test. The relationship between categorical variables was evaluated with chi-square tests. Relationships between variables were evaluated with the Spearman correlation analysis. A value of two-sided *p* < 0.05 was considered statistically significant.

## 3. Results

A total of 1006 questionnaires were gathered, among which 39 were collected manually and inserted, with the rest gathered electronically. One month was a timeline determined not by the authors prior to the survey conduction but from the subjects’ response rate. The responses had highs and lows during the month followed by a week with a complete lack of new responses to the questionnaire, and the authors declared the questionnaire closed afterwards. Patient age, gender, systemic disease, smoking, visits to the dentist, and awareness of the existence of periodontitis as an oral disease are summarized in Table 1. Regarding the term “periodontitis”, half of the participants reported having not ever heard it. Females were more informed than males (*p* < 0.001).

### 3.1. Toothbrushing

Among the 1006 completing the questionnaire, 100% of them reported toothbrushing daily. The most frequently used toothbrush among the population was the manual toothbrush (91.1%) over the electric toothbrush (8.9%). The use of electric toothbrushes was statistically more prevalent in males (12.6% M, 7.9% F, *p* = 0.036). Most (71%) of the toothbrushes used were of medium hardness bristles, and only 2.7% reported using a hard-bristle toothbrush. Among the hard-bristle users, females were statistically more prevalent than males (31% F, 1.6% M, *p* = 0.002). The toothbrush was changed by 44.8 % of the population more frequently than every 3 months; however, 54.4% used the same toothbrush for more than 3 months in a row. The circular movement of the toothbrush was the most preferred (36.7%), although horizontal (14.2%), vertical (12.5%), bushing from the gingiva to the tooth (Bass technique) (9.1%), and a combination of different movements (27.6%) were reported as well. Among females, the most prevalent technique was circular brushing (40.3% F, 24.9% M, *p* < 0.001), whereas among males, a combination of different movements was prevalent (33.1% M, 25.9% F, *p* < 0.001). Almost sixty-six percent of the population reported toothbrushing twice daily, followed by 25% toothbrushing once daily and a small portion (8.5%) performing tooth cleaning 3–4 times a day. The duration of toothbrushing was 2–3 min in 72% of the population, which was comparable between males and females. Only 8% of the population performed toothbrushing immediately after consuming a list of fruits or fruit juice containing citric acid (Table 2).

Overall, 99% of the population used a toothbrush in combination with commercially available toothpaste. The reported toothpaste used varied from standard use to toothpaste for gingival inflammation, tooth whitening, and dentinal hypersensitivity.

### 3.2. Interdental Instruments

Interdental cleaning was performed by 77.1% of the population, mostly using dental floss (67.4%) and mainly once daily (52.2%). Bleeding immediately after interdental instrument use was reported in 39.2% of the population that used it (Table 2). The use of interdental instruments was comparable between genders (21.9% of females vs. 25.9% of males, *p* = 0.2). However, 55% of females used them once daily compared to 44% of males, the difference being statistically significant (*p* = 0.016).

### 3.3. Mouthwash

Overall, 41.5% of the population answering the questionnaire reported using commercially available mouthwash suggested for the prevention or improvement of gingival inflammation, the reinforcement of oral hygiene, or halitosis (Table 2). The use was reported once daily (67%) or even more frequently (33%). The use of mouthwash was statistically more prevalent among females (44.2% F, 33.6% M, *p* = 0.004).

More detailed statistical analyses were added as Supplementary Materials (Tables S1–S4), while the summarized results are presented in the tables inserted in the manuscript (Table 1 and Table 2).

## 4. Discussion

The aim of the present survey was to evaluate the quality of information that patients in Albania have on oral hygiene performance in order to evidence what are the parts to be improved during the instruction and promotion of oral hygiene. The scrolling of each component of oral hygiene instructions investigated by the questionnaire is discussed with the purpose of gathering the evidence that could be included when communicating the oral hygiene instructions to the patient based on the gaps the present population demonstrated.

The data were collected electronically and manually; however, the difference in the number between manual and electronically completed questionnaires was so big that a comparison could not be made. The difference between the rate of manual and electronically completed questionnaires is a reflection of the help that technology can provide, in this case for the research and, overall, for everyday life. Technology overcomes the obstacles that distance and time can present and makes it possible to respect the differences, sometimes impeding different schedules between operators and study subjects. In spite of the usefulness of technology for the aim of the present study, direct communication with patients provides more detailed and complete information.

The results showed that all patients brushed their teeth daily, with the most prevalent frequency being twice daily (66.5%), with a duration of 2–3 min in 72% of the cases, and 77.1% of them were using interdental instruments along with toothbrushing. A study of 372 12-year-old children clinically examined for their dental status who completed an oral health behavior questionnaire reported that only 67% brushed their teeth daily [19]. Considering the present study size and age population, there could be a limit of comparison; however, in more than 11 years of difference between studies, an improvement towards the integration of daily toothbrushing could be noticed. Overall, the information that the most prevalent part of the population has is in accordance with the worldwide accepted evidence on oral hygiene [24]. However, for every component of oral hygiene investigated through the questionnaire, there was a proportion of the population that did not have correct information on the type of instrument and the proper use of each one. This specific point of misinformation could be addressed and emphasized in the oral hygiene training of dental professionals along with the need for oral hygiene instructions to be communicated in an individually tailored way [26].

Up to now, it is unclear how much dental plaque has to be eliminated in order to prevent periodontal inflammation [27]; however, in the individual risk evaluation of the patient, the target for the full mouth plaque score (FMPS) is set at less than 25% [28]. The evidence does not show any significant difference between manual and oscillating–rotating toothbrushes; however, for specific tooth surfaces, for example, lingual surfaces of the mandibular incisors or posterior teeth, cleaning with electric toothbrushes was found to be more effective [29]. In the present study, 91.1% of the cases used manual toothbrushes. The authors assume that the low percentage of electric toothbrush use could be influenced mostly by the price, but it might be related to the lack of presentation from the dentist during instructions as well. Thus, the oral hygiene information provided in the present country could benefit from adding electric toothbrushes for specific areas in case the patient has difficulties in plaque removal and can financially afford it. The choice between a manual and electric toothbrush should be individually tailored but always given with instructions and the monitoring of use in order to prevent side effects related not directly to the toothbrush but when it is used with force, carelessly damaging the hard dental tissues and soft tissues [30]. Among the present population, 2.7% reported using hard toothbrushes. Hard toothbrushes have been demonstrated to be associated with soft tissue [31] and dental abrasion [32]. Furthermore, the technique of toothbrushing was noticed to be incorrect in patients reporting vertical (12.5%), horizontal (14.2%), or combined (27.6%) toothbrush movement. Evidence suggests that plaque control is better obtained through the Fones or Bass technique [33], which is evidence that is necessary to be included in oral hygiene instruction.

Regarding the replacement of a toothbrush, 54.4% of the population reported longer than a 3-month use of the toothbrush. Toothbrush replacement is recommended due to toothbrush wear and bristle deformation, reducing the effectiveness of plaque removal [34], and, secondly, due to microbiological contamination after three months at the latest and in the best case after one or two months [35]. The authors recommend informing the patients to change their toothbrushes at least every 3 months and immediately after professional dental plaque removal.

The most common frequency of toothbrushing in the present population was twice daily, which is in accordance with the generally recommended frequency based on self-reported infrequent brushers, demonstrating higher incidences and increments of carious lesions than frequent brushers [36]. On the other hand, more frequent and shorter brushing times reported in a part of the population may increase the risk of adverse effects [37]. From the authors’ experience, subtle and compassionate communication, including the above-mentioned information, could be introduced, particularly to patients self-reporting overly brushing who have not received clinical excellence when dentists examine them for their oral hygiene because the sense of frustration created might reduce their compliance. However, both sides of infrequent and overfrequent brushers should be communicated as part of the instructions for oral hygiene.

With respect to toothbrushing duration, the most reported interval was 2–3 min. It has been shown that a 2 min brushing time is effective enough for plaque reduction without the risk of demotivating the patients with longer time [38,39]. However, the authors emphasize that the communication of the time should be inclusive of interdental instrument use and other oral hygiene practices advised based on the specific clinical case so that misunderstandings of the 2 min brushing time. 

Overall, the entire population used dentifrices. The additional effect of dentifrice on toothbrushing alone has been shown to be insignificant for a reduction in plaque [40]; however, it is used for the delivery of agents for caries prevention, anti-inflammatory agents, abrasive agents for tooth whitening, etc. All these additive agents have their respective side effects, which the authors judge are useful to share with the patients in order to properly guide the selections and use of the toothpaste, which should be evidence based and not based on the description of the paste and what the commercials put in their evidence. Evidence shows that triclosan use is related to the damage of the equilibrium of the oral microbiota [41]; stannous fluoride used for caries prevention has been shown to pigment the teeth [42]; whitening agents are abrasive agents that have not been studied for their abrasive side effects [43] and are potentially more damaging than baking soda [44]; and sodium lauryl sulfate (SLS) presence affects aphthous ulcers in a way that when the toothpaste is free of SLS, the duration of ulcers and the mean pain score were significantly decreased [45].

Overall, 41.5% of the included population reported using mouth rinse. Although mouth rinses have demonstrated a higher impact on the prevention of gingival inflammation development and in plaque level control compared to dentifrices, dentifrices may be the ideal delivery format for the general population, and mouth rinse could be more suitable for individuals at higher risk or in specific clinical scenarios [44]. The authors suggest this information and the promotion of limited mouth rinse use be inserted in the oral hygiene instructions.

Daily interproximal cleaning is essential for maintaining interproximal gingival health and preventing dental caries in this location [46]. Interdental instruments include dental floss, interdental brushes, woodsticks, and oral irrigators. The interdental space is an important determinant in choosing the type of instrument [47]. In the present population, the most used instrument was dental floss, with almost 39.2% of people experiencing bleeding upon using interdental instruments. Even though bleeding per se is not a reason for cessation of interdental cleaning but an indicator of inflammation that needs to be treated by interdental cleaning [48], the patient can cause bleeding due to the inappropriate instruments used. Dental floss is more appropriate for teeth that are very adjacent to each other, and despite the lack of evidence, a more lubricious/waxed dental floss can be passed over the approximal contact with less pressure to reduce the risk of papillary trauma [49]. When the tooth is periodontally compromised, the attachment loss exposes the concavities in the approximal root surfaces, and interdental brushes are the most recommended instruments for removing plaque as far as 2–2.5 mm below the gingival margin [50]. Among the interdental toothbrushes, the one that is cylindrical shaped is more effective than conical-shaped and straight ones, which are more effective than angled ones [51,52,53]. Other instruments are oral irrigators designed to flush away loosely adherent plaque through the mechanical action of a stream of water [30]. The literature does not support the use of woodsticks [37]. The myriad of instrument types and indications should be communicated to the patient after specific interdental space evaluation of the different areas of the dentition and revised routinely as long as the interdental space could be subject to changes in time in order for the patient to have the correct instruments for each area and use them without inflicting any self-damage, more specifically bleeding upon interdental flossing.

A limitation of the present survey is the fact that it has provided demographic data but lacks depth in analysis. Detailed analysis of the influence of socioeconomic status, education level, and access to dental care on the oral hygiene information exposed by the present population was not the aim of the survey. The aim was to observe if the information that reached the population was correct and where the pitfalls were that needed intervention. Future and more detailed clinical surveys on oral hygiene in Albania could profit from the findings of the present study and emphasize limits. Oral examination was not the aim of the present study, and the lack of information on how self-reported oral hygiene was reflected in the oral condition with the presence of dental plaque and periodontal health represents a limit, but it should be considered for future studies as a subsequent step.

## 5. Conclusions

Within the limits of this study, it could be concluded that the present population seemed to be mostly appropriately informed, but the information needs further revision and promotion by dentists with the need for further extension with training on this topic, emphasizing the information that the present population had but where not appropriate, like the use of hard bristle toothbrush, the use of vertical and horizontal movements of the toothbrush, the use of a toothbrush for longer than 3 months, the use of a woodstick, the forced use of interdental instruments that can cause bleeding upon their use, and the use of mouth rinse when it is not necessary. However, future clinical studies could determine the appropriateness of execution and the grade of individually tailored modification of the information each subject has.

## Figures and Tables

**Table 1:** Patient age, gender, systemic disease, smoking, visits to the dentist, and knowledge of periodontitis.

Patient Information	
Age Mean ± SD Median (IQR)	33 ± 11.3 32 (13)
Gender	Male (24.8%) Female (75.2%)
City	Tirana (63.7%) Other cities (36.3%)
Systemic conditions	Yes (5.4%) No (94.6%)
Smoking	Yes (20.2%) No (79.8%)
Visits to the dentist	1/year (55%) 2/year (36%) 3/year (8%) When in pain or other symptoms (1%)
Awareness of periodontitis as an oral disease	Yes (49.1%) No (50.9%)

**Table 2:** Instruments for the mechanical removal of plaque, toothbrush and interdental instrument characteristics, and the use of toothpaste and mouthwash.

**Type of toothbrush**	Reporting toothbrushing (100% of the population) Manual (91.1%) Electric (8.9%)
Hardness of toothbrush bristles	Soft (26.3%)
	Medium (71%)
	Hard (2.7%)
Technique of toothbrushing	Horizontal (14.2%)
	Vertical (12.5%) Circular (36.7%) Brushing from gingiva to tooth (9.1) Combination (27.6%)
Frequency of toothbrushing	1/day (25%)
	2/day (66.5%)
	3/day (8%)
	4/day (0.5%)
Duration of toothbrushing	≤2 min (17.7%) 2–3 min (72%) ≥3 min (10.3%)
Frequency of toothbrush changing	≤3 times/year (54.4%) 4 times/year (0.8%) ≥5 times/year (44.8%)
Toothbrushing immediately after the consumption of citric-acid-containing fruits/fruit juice	Yes (8%) No (92%)
**Use of interdental instruments**	Yes (77.1%) No (22.9%)
Type of interdental instruments	Dental floss (67.4%) Interdental brushes (7.4%) Woodstick (15.1%) Dental floss and interdental brushes (2.5%) Dental floss and woodstick (6.2%) All (1.4%)
Frequency of interdental instruments use	>1 time/day (47.8%) ≤1/day (52.2%)
Bleeding once interdental instruments used	Yes (39.2%) No (60.8%)
**Toothpaste use**	Yes (98.6%) No (1.4%)

## Data Availability

Data are unavailable due to privacy and ethical restrictions.

## Supplementary Materials

The following supporting information can be downloaded at https://www.mdpi.com/article/10.3390/dj13010001/s1, Table S1: Geographical distribution and influence on parameters taken; Table S2: Gender influence on the evaluated parameters; Table S3: Age influence on the evaluated parameters; Table S4: Correlation coefficients age, gender, city, and variables. *rho*: Spearman correlation coefficient.
