# Supplementary material for: Self-Reported Oral Hygiene Performance of Patients in Albania: A Questionnaire-Based Survey
Source: Dent J (Basel). 2024 Dec 24;13(1):1. doi: 10.3390/dj13010001 (PMC11763437; doi:10.3390/dj13010001)
Supplement: Supplementary file 1 [file dentistry-13-00001-s001.zip › Table S1 Geografical distribution and influence on parameters taken.pdf]

Table S1. Geographical distribution and influence on parameters taken.

[+]Pearson Chi-Square test; [*] Continuity Correction Yates Chi-Square test

[++] Mann Whitney *U* test; [+]Pearson Chi-Square test; [*] Continuity Correction Yates Chi-Square test

Variables	Tirana *n (%)*	Other cities *n (%)*	*p* value
Age			**0,010**[++]
Mean±SD	33,14±10,94	31,86±11,84	
Median (*IQR*)	32 (12) *a*	30 (15) *b*	
Gender			**0,002**[+]
Female	499 (78,3) *a*	250 (69,6) *b*	
Male	138 (21,7) *a*	109 (30,4) *b*	
Smoking			0,295[+]
No	499 (78,8)	297 (81,6)	
Yes	134 (21,2)	67 (18,4)	
Systemic diseases			0,361[*]
No	597 (94,0)	347 (95,6)	
Yes	38 (6,0)	16 (4,4)	
Type of toothbrush			0,757[+]
Manual	585 (91,3)	331 (90,7)	
Electric	56 (8,7)	34 (9,3)	
Technique of toothbrushing			0,129[+]
Vertical movement	72 (11,3)	53 (14,6)	
Horizontal movement	84 (13,1)	58 (16,0)	
Circular movement	236 (36,9)	131 (36,2)	
Brushing from gingival to tooth	56 (8,8)	35 (9,7)	
More than one	191 (29,9)	85 (23,5)	
Duration of toothbrushing			**0,015**[+]
Less than 2 minutes	117 (18,3) *a*	60 (16,6) *a*	
2-3 minutes	470 (73,4) *a*	251 (69,3) *a*	
More than 3 minutes	53 (8,3) *b*	51 (14,1) *a*	
Frequency of toothbrushing during the day			0,480[+]
1 time	152 (23,8)	99 (27,3)	
2 times	436 (68,2)	230 (63,4)	
3 times	48 (7,5)	32 (8,8)	
4 times	3 (0,5)	2 (0,6)	
Frequency of toothbrush changing during the year			**0,010**[+]
Once a year	34 (5,3) *b*	34 (9,4) *a*	
Twice a year	124 (19,5) *a*	58 (16,1) *a*	
3 times a year	173 (27,2) *b*	120 (33,2) *a*	
4 times a year	4 (0,6) *a*	4 (1,1) *a*	

More often 5	302 (47,4) [b]	145 (40,2) [a]	
Use of interdental instruments			0,236[+]
Yes	138 (21,7)	91 (24,9)	
No	499 (78,3)	274 (75,1)	
Type of interdental instrument			**0,025**[+]
Interdental floss	334 (67,3) [a]	184 (67,4) [a]	
Interdental brush	34 (6,9) [a]	23 (8,4) [a]	
Stick	67 (13,5) [a]	49 (17,9) [a]	
Floss and brush together	18 (3,6) [b]	1 (0,4) [a]	
Floss and stick	36 (7,3) [a]	12 (4,4) [a]	
All three	7 (1,4) [a]	4 (1,5) [a]	
Variables	Tirana<br>*n (%)*	Other cities<br>*n (%)*	*p* value
Frequency of interdental instrument use			0,540[+]
Once a day	263 (53,0)	140 (69,6)	
Less than once a year	233 (47,0)	109 (30,4)	
Bleeding when interdental instrument use			0,109[+]
No	315 (62,9)	155 (57,0)	
Yes	186 (37,1)	117 (43,0)	
Use of toothpaste			0,807[*]
No	8 (1,3)	6 (1,7)	
Yes	631 (98,7)	356 (98,3)	
Use of mouth rinse			0,822[+]
No	373 (58,7)	210 (58,0)	
Yes	262 (41,3)	152 (42,0)	
Toothbrushing after eating fruits			**0,030**[*]
No	592 (93,5) [a]	321 (89,4) [b]	
Yes	41 (6,5) [a]	38 (10,6) [b]	
Have you ever heard of periodontitis			0,175[+]
No	313 (49,3)	192 (53,8)	
Yes	322 (50,7)	165 (46,2)	
Toothbrush bristle hardness			**0,002**[+]
Soft	191 (29,8) [a]	73 (20,1) [b]	
Medium	431 (67,2) [a]	282 (77,7) [b]	
Hard	19 (3,0) [a]	8 (2,2) [a]	
