# Supplementary material for: Self-Reported Oral Hygiene Performance of Patients in Albania: A Questionnaire-Based Survey
Source: Dent J (Basel). 2024 Dec 24;13(1):1. doi: 10.3390/dj13010001 (PMC11763437; doi:10.3390/dj13010001)
Supplement: Supplementary file 1 [file dentistry-13-00001-s001.zip › Table S2 Gender influence.pdf]

Table S2. Gender influence on the evaluated parameters.

[++] Mann Whitney *U* test; [+]Pearson Chi-Square test; [*] Continuity Correction Yates Chi-Square test

[+]Pearson Chi-Square test; [*] Continuity Correction Yates Chi-Square test; [**] Fisher's Exact test

Variables	Female *n (%)*	Male *n (%)*	*p* value
Age			**0,001**[++]
Mean±SD	32,15±11,14	34,41±11,43	
Median (*IQR*)	30 (14) [a]	35 (9) [b]	
Smoking			**<0,001**[+]
No	637 (85,6) [a]	154 (62,3) [b]	
Yes	107 (14,4) [a]	93 (37,7) [b]	
Systemic diseases			0,783[*]
No	704 (94,4)	233 (95,1)	
Yes	42 (5,6)	12 (4,9)	
Type of toothbrush			**0,036**[*]
Manual	690 (92,1) [a]	216 (87,4) [b]	
Electric	59 (7,9) [a]	31 (12,6) [b]	
Technique of toothbrushing			**<0,001**[+]
Vertical movement	98 (13,1) [a]	26 (10,6) [a]	
Horizontal movement	89 (11,9) [a]	53 (21,6) [b]	
Circular movement	301 (40,3) [a]	61 (24,9) [b]	
Brushing from gingival to tooth	65 (8,7) [a]	24 (9,8) [a]	
More than one	193 (25,9) [a]	81 (33,1) [b]	
Duration of toothbrushing			**0,011**[+]
Less than 2 minutes	122 (16,4) [a]	54 (21,9) [b]	
2-3 minutes	536 (71,8) [a]	178 (72,1) [a]	
More than 3 minutes	88 (11,8) [a]	15 (6,1) [b]	
Frequency of toothbrushing during the day			**<0,001**[+]
1 time	146 (19,6) [a]	104 (42,1) [b]	
2 times	529 (70,9) [a]	131 (53,0) [b]	
3 times	69 (9,2) [a]	9 (3,6) [b]	
4 times	2 (0,3) [a]	3 (1,2) [a]	
Frequency of toothbrush changing during the year			**0,008**[+]
Once a year	41 (5,5) [a]	27 (10,9) [b]	
Twice a year	128 (17,2) [a]	54 (21,9) [a]	
3 times a year	220 (29,6) [a]	66 (26,7) [a]	
4 times a year	5 (0,7) [a]	3 (1,2) [a]	
More often 5	349 (47,0) [a]	97 (39,3) [b]	
Use of interdental instruments			0,191[+]
Yes	163 (21,9)	64 (25,9)	

Variables	Female *n* (%)	Male *n* (%)	*p* value
No	582 (78,1)	183 (74,1)	
Type of interdental instrument			**<0,001**[+]
Interdental floss	411 (71,2) [a]	103 (56,0) [b]	
Interdental brush	49 (8,5) [a]	8 (4,3) [a]	
Stick	65 (11,3) [a]	49 (26,6) [b]	
Floss and brush together	15 (2,6) [a]	4 (2,2) [a]	
Floss and stick	28 (4,9) [a]	18 (9,8) [b]	
All three	9 (1,6) [a]	2 (1,1) [a]	
Frequency of interdental instrument use			**0,016**[+]
Once a day	320 (55,0) [a]	82 (44,8) [b]	
Less than once a year	262 (45,0) [a]	101 (55,2) [b]	
Bleeding when interdental instrument use			0,385[+]
No	348 (59,8) [a]	116 (63,4) [a]	
Yes	234 (40,2) [a]	67 (36,6) [a]	
Use of toothpaste			0,125[**]
No	8 (1,1)	6 (2,4)	
Yes	737 (98,9)	240 (97,6)	
Use of mouth rinse			**0,004**[+]
No	415 (55,8)	162 (66,4)	
Yes	329 (44,2)	82 (33,6)	
Toothbrushing after eating fruits			0,563[*]
No	676 (91,6)	227 (93,0)	
Yes	62 (8,4)	17 (7,0)	
Have you ever heard of periodontitis			**<0,001**[+]
No	349 (47,2) [a]	151 (62,4) [b]	
Yes	391 (52,8) [a]	91 (37,6) [b]	
Toothbrush bristle hardness			**0,002**[+]
Soft	216 (28,9) [a]	46 (18,6) [b]	
Medium	508 (68,0) [a]	197 (79,8) [b]	
Hard	23 (3,1) [a]	4 (1,6) [a]	
