# Supplementary material for: Self-Reported Oral Hygiene Performance of Patients in Albania: A Questionnaire-Based Survey
Source: Dent J (Basel). 2024 Dec 24;13(1):1. doi: 10.3390/dj13010001 (PMC11763437; doi:10.3390/dj13010001)
Supplement: Supplementary file 1 [file dentistry-13-00001-s001.zip › Table S3 Age influence on the parameters evaluated.pdf]

Table S3. Age influence on the evaluated parameters.

[+] Mann Whitney *U* test; [++] Kruskal Wallis test

Variables	*n*	Age *Mean±SD*	Age *Median* (*IQR*)	*p* value
Smoking				0,414[+]
No	561	32,28±10,35	32 (14)	
Yes	147	31,73±8,39	31 (9)	
Systemic diseases				**<0,001**[+]
No	678	31,75±9,28	31 (12)	
Yes	30	41,47±17,90	43 (30)	
Type of toothbrush				0,315[+]
Manual	643	32,12±10,02	31 (12)	
Electric	65	32,63±9,57	34 (8)	
Technique of toothbrushing				**0,001**[++]
Vertical movement (1)	79	35,46±11,92	35 (16)	1-3: **0,002**
Horizontal movement (2)	88	31,77±9,08	31 (11)	3-5: **0,036**
Circular movement (3)	279	31,05±8,50	30 (11)	
Brushing from gingival to tooth (4)	64	31,92±12,82	34 (21)	
More than one (5)	198	32,67±10,17	33 (13)	
Duration of toothbrushing				**0,004**[++]
Less than 2 minutes (1)	114	32,76±10,53	33 (10)	1-3: **0,003**
2-3 minutes (2)	523	32,36±9,58	32 (12)	2-3: **0,001**
More than 3 minutes (3)	71	29,75±11,61	29 (16)	
Frequency of toothbrushing during the day				**<0,001**[++]
1 time	174	33,42±9,87	34 (10)	1-3: **<0,001**
2 times	470	32,16±9,89	31 (13)	2-3: **0,028**
3 times	61	28,67±10,42	26 (15)	
4 times	3	31,00±7,00	34 (6,5)	
Frequency of toothbrush changing during the year				**<0,001**[++]
Once a year	50	29,48±11,61	30 (16)	1-2: **0,020**
Twice a year	120	34,08±10,10	35 (12)	2-3: **0,035**
3 times a year	215	33,61±10,45	33 (12)	2-5: **<0,001**
4 times a year	5	37,20±14,22	33 (21)	
More often 5	318	30,81±8,96	30 (13)	
Use of interdental instruments				-
Yes	-	-	-	
No	708	32,16±9,97	31 (12)	
Type of interdental instrument				0,423[++]
Interdental floss	477	32,14±9,35	31 (12)	
Interdental brush	50	32,08±13,76	33 (21)	
Stick	106	31,58±11,37	31 (14)	

Floss and brush together	19	30,79±9,27	31 (18)	
Floss and stick	46	34,11±8,32	35,5 (10)	
All three	10	33,50±10,46	36 (20)	

Variables	*n*	Age<br>*Mean±SD*	Age<br>*Median* (*IQR*)	*p* value
Frequency of interdental instrument use				**0,015**$^{+}$
Once a day	370	33,12±9,67	32 (12)	
Less than once a year	338	31,12±10,21	30 (13)	
Bleeding when interdental instrument use				0,552^{+}
No	430	32,57±10,26	31 (13)	
Yes	278	31,53±9,49	31 (12)	
Use of toothpaste				0,281^{+}
No	11	25,09±12,09	29 (18)	
Yes	697	32,28±9,91	31 (12)	
Use of mouth rinse				0,870^{+}
No	392	32,03±10,02	33 (12)	
Yes	316	32,33±9,93	31 (12)	
Toothbrushing after eating fruits				**0,010**$^{+}$
No	654	32,17±9,58	32 (12)	
Yes	54	32,04±13,97	29,5 (22)	
Have you ever heard of periodontitis				**<0,001**$^{+}$
No	325	30,82±10,39	30 (14)	
Yes	383	33,30±9,47	33 (11)	
Toothbrush bristle hardness				0,122^{++}
Soft	188	31,55±9,47	30 (13)	
Medium	501	32,56±10,15	32 (13)	
Hard	19	27,79±9,41	27 (13)	
