# Supplementary material for: Self-Reported Oral Hygiene Performance of Patients in Albania: A Questionnaire-Based Survey
Source: Dent J (Basel). 2024 Dec 24;13(1):1. doi: 10.3390/dj13010001 (PMC11763437; doi:10.3390/dj13010001)
Supplement: Supplementary file 1 [file dentistry-13-00001-s001.zip › Table S4 Correlation coefficients age, gender, city, and variables.pdf]

Table S4. Correlation coefficients age, gender, city, and variables. *rho*: Spearman correlation coefficient

Variables		Age	Gender	City
**Type of toothbrush**	*rho*	0,032	0,070	-0,01
	p value	0,316	**0,026**	0,757
**Technique of toothbrushing**	*rho*	**0,007**	**0,027**	0,077
	p value	0,835	0,403	**0,014**
**Duration of toothbrushing**	*rho*	-0,075	-0,092	-0,067
	p value	**0,017**	**0,004**	**0,035**
**Frequency of toothbrushing during the day**	*rho*	-0,119	-0,217	**0,022**
	p value	**<0,001**	**<0,001**	0,495
**Frequency of toothbrush changing during the year**	*rho*	-0,114	-0,094	0,059
	p value	**<0,001**	**0,003**	0,061
**Use of interdental instruments**	*rho*	0,036	-0,042	0,037
	p value	0,260	0,192	0,236
**Type of interdental instrument**	*rho*	0,010	0,151	0,017
	p value	0,789	**<0,001**	0,635
**Frequency of interdental instrument use**	*rho*	-0,088	0,087	-0,022
	p value	**0,015**	**0,016**	0,54
**Bleeding when interdental instrument use**	*rho*	-0,022	-0,031	-0,058
	p value	0,552	0,386	0,110
**Use of toothpaste**	*rho*	0,034	-0,050	0,017
	p value	0,282	0,116	0,600
**Use of mouth rinse**	*rho*	-0,005	-0,093	-0,007
	p value	0,870	**0,003**	0,822
**Toothbrushing after fruits consumption**	*rho*	-0,082	-0,023	-0,073
	p value	**0,010**	0,476	**0,022**
**Have you ever heard of periodontitis**	*rho*	0,122	-0,131	0,043
	p value	**<0,001**	**<0,001**	0,175
**Toothbrush hardness**	*rho*	0,036	0,085	-0,093
	p value	0,254	**0,008**	**0,003**
